# Revisiting gut microbiota-driven ammonia metabolism: from disease burden to physiological adaptation

**DOI:** 10.1080/19490976.2026.2722486

**Published:** 2026-08-25

**Authors:** Hao Chen, Zhen Wang, Jing Huang, Zheng Yu

**Affiliations:** a Human Microbiome and Health Group, Department of Microbiology, Xiangya School of Basic Medical Sciences, Central South University, Changsha, China; b Human Microbiome and Health Group, Department of Parasitology, Xiangya School of Basic Medical Sciences, Central South University, Changsha, China

**Keywords:** Gut microbiota, ammonia metabolism, nitrogen homeostasis, ammonia burden, physiological adaptation

## Abstract

Ammonia homeostasis is governed by interconnected host and microbial pathways, including hepatic ureagenesis, glutamine synthesis, and gut microbiota-mediated urea hydrolysis, proteolysis, and amino acid deamination. Ammonia has historically been viewed primarily as a nitrogenous waste product and neurotoxic molecule, a concept strongly supported by studies of hyperammonemia and hepatic encephalopathy. Impaired hepatic clearance, portosystemic shunting, and enhanced gut-derived ammonia input increase systemic ammonia burden and are linked to neurological dysfunction, muscle wasting, immune dysregulation, and progression of liver disease. In the gut lumen and mucosal environment, however, gut microbes not only generate ammonia but can also reuse ammonia-derived nitrogen for amino acid synthesis, microbial biomass formation, and community nitrogen exchange. Evidence from selected physiological and preclinical models indicates that microbiota-derived ammonia may contribute to nitrogen recycling, microbial community maintenance, enteric neural regulation, or metabolic adaptation under defined conditions. These roles are more context-specific and less broadly established than the pathological effects of systemic hyperammonemia. When epithelial barrier integrity is compromised, hepatic clearance declines, or host buffering reserves are depleted, elevated ammonia can increase epithelial exposure, portal ammonia input, or peripheral blood ammonia. Conversely, evidence that reduced microbiota-derived ammonia contributes to disease remains limited and currently comes mainly from selected animal models. This review re-examines ammonia metabolism from a gut microbiota-centered perspective. We discuss the ecological origins, spatial distribution, exposure characteristics, host-interface effects, and context-specific outcomes of microbiota-derived ammonia in physiology and disease, and discuss how ammonia should be measured, stratified, and targeted in microbial nitrogen metabolism.

## Graphical abstract

**Figure:**
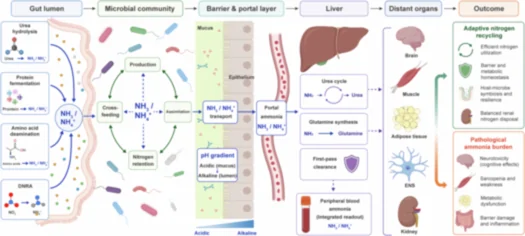

## 1 Introduction

Ammonia is a fundamental molecule in mammalian nitrogen metabolism. It has traditionally been viewed as a waste product generated during catabolism and cleared by hepatic detoxification. Ammonia has commonly been considered a nitrogenous terminal product generated by amino acid deamination, nucleotide degradation, and microbial urease reactions. Because ammonia has well-established neurotoxic potential, host systems constrain ammonia accumulation through hepatic ureagenesis and glutamine synthesis, thereby limiting injury to the brain and other organs.^1^ ^,^ ^2^ This view derives primarily from studies of hyperammonemia and hepatic encephalopathy. In these disease settings, elevated ammonia is closely associated with astrocyte swelling, disruption of the cerebral glutamate‒glutamine cycle, oxidative stress, mitochondrial dysfunction, and cerebral edema.^3^ ^,^ ^4^ Within the traditional host metabolic framework, ammonia has therefore long been viewed as a metabolic waste product and neurotoxic molecule requiring rapid clearance.

However, advances in host-microbiota research indicate that defining ammonia solely as a toxic end product is insufficient to explain how gut microbes produce, reuse, and transfer ammonia-derived nitrogen. Ammonia in the intestinal tract does not arise from a single homogeneous gut compartment. Instead, it is shaped by site-specific host and microbial processes, including epithelial glutamine metabolism, host-derived urea delivery, microbial urea hydrolysis, proteolysis, and amino acid deamination.^5^ ^,^ ^6^ Selected community members participate in urea-nitrogen salvage and ammonia assimilation, reincorporating free nitrogen into amino acids and microbial biomass.^6^ ^,^ ^7^ In experimental models, inhibition of gut microbial urease can reduce host ammonia burden. In contrast, evidence that insufficient microbiota-derived ammonia can impair brain glutamine availability currently comes mainly from a chronic stress mouse model and should be interpreted as model-specific.^8^ ^,^ ^9^ Thus, current evidence supports a clear distinction: pathological effects of elevated systemic ammonia are well established in clinical and experimental settings, whereas disease-related effects of reduced microbiota-derived ammonia remain more limited and preclinical.

A spatial distinction is important from the outset. The ileum and ascending or proximal colon represent two major but functionally different sites of host-microbial nitrogen exchange. The ileum is characterized by rapid transit, active host absorption and secretion, epithelial glutamine metabolism, and direct access to portal drainage. Urea delivered across the ileal epithelium may therefore provide substrate for urease-positive microbes before dense colonic fermentation becomes dominant. The ascending or proximal colon, in contrast, contains a denser microbial community, receives residual dietary and host-derived substrates, and supports active fermentation, urea hydrolysis, ammonia assimilation, and nitrogen cross-feeding.^10^ ^-^ ^12^ Urea delivery into the intestinal lumen should therefore be treated as spatial substrate delivery rather than merely as terminal host waste disposal.

Ammonia metabolism is embedded in cross-organ physiological networks. Liver-centered studies have shown that, in cirrhosis and hepatic encephalopathy, hyperammonemia reflects the combined effects of increased intestinal ammonia generation, portosystemic shunting, impaired hepatic detoxification, and increased extrahepatic buffering demand, rather than a single expanded source.^1^ ^,^ ^13^ ^,^ ^14^ The kidney regulates ammonia release and excretion under physiological and pathological conditions, skeletal muscle functions as a peripheral buffer through glutamine synthesis, and brain tissue is highly sensitive to ammonia-induced metabolic and osmotic stress.^2^ ^,^ ^13-15^ Thus, ammonia is not handled by the liver alone but moves across ileal, colonic, portal, hepatic, systemic, and distal-organ compartments, where it is transported, cleared, reassimilated, or retained.

This review addresses four mechanistic questions: which microbial pathways generate or assimilate ammonia; where ammonia first accumulates across ileal, ascending-colonic, distal-colonic, portal, and systemic compartments; how epithelial transport and hepatic clearance determine host exposure; and which host conditions convert compartment-specific ammonia production or exposure into adaptive nitrogen recycling or pathological burden. From a gut microbiota-centered perspective, total peripheral blood ammonia is an integrated readout generated after luminal production, community assimilation, trans-barrier transport, portal input, and hepatic processing; it does not fully capture local exposure or tissue-specific effects. Similar peripheral ammonia concentrations may reflect distinct ileal or colonic exposures, portal inputs, and organ-processing burdens.^2^ ^,^ ^13^ ^,^ ^16^ Local ammonia changes, even in the absence of systemic hyperammonemia, may be amplified into pathological signals when epithelial barrier injury, enhanced transepithelial transport, portosystemic shunting, or reduced host buffering capacity is present.^1^ ^,^ ^13^ ^,^ ^14^ Evidence from early-life nutrition, nutritional adaptation, and selected preclinical models suggests that microbiota-derived ammonia can support nitrogen recycling or specific host responses under defined conditions.^9^ ^,^ ^17^ ^,^ ^18^ However, evidence that insufficient ammonia input causes disease remains limited and should not be treated as equivalent to the clinical evidence for hyperammonemia. On this basis, we first review the ecological foundations of gut microbiota-derived ammonia, then discuss ileal-colonic and extraintestinal host interfaces that sense and reshape this metabolite, compare its context-specific outcomes in physiology and disease, and finally address measurement strategies, knowledge gaps, and translational opportunities.

## 2 Ecological foundations of gut microbial ammonia metabolism

### 2.1 Multiple microbial routes converge on ammonia generation

The gastrointestinal tract contributes substantially to portal ammonia input, but this contribution is not anatomically uniform. The ileum and ascending or proximal colon are especially relevant because they combine microbial activity with access to host-derived nitrogen substrates. In the ileum, epithelial glutamine metabolism, host-derived urea delivery, rapid absorption, and portal drainage can make locally generated ammonia rapidly available to host metabolism.^1^ ^,^ ^19^ ^,^ ^20^ In the ascending and proximal colon, higher microbial density, slower transit, fermentation of residual substrates, and urea hydrolysis create a distinct compartment for microbial ammonia production, assimilation, and nitrogen exchange.^1^ ^,^ ^19^ ^,^ ^21^ Culture-based and microbiological studies have identified several gut-associated ammonia-generating taxa, including *Escherichia coli*, *Bacteroides* spp., *Proteus* spp., *Clostridium* spp., and *Peptostreptococcus* spp. These organisms can continuously replenish the luminal ammonia pool through urease-dependent urea hydrolysis, proteolysis and deaminative fermentation, and transformation of other nitrogen-containing substrates.^1^ ^,^ ^21^ Methanogenic archaea may also contribute to intestinal ammonia generation.^1^

**Figure 1.**
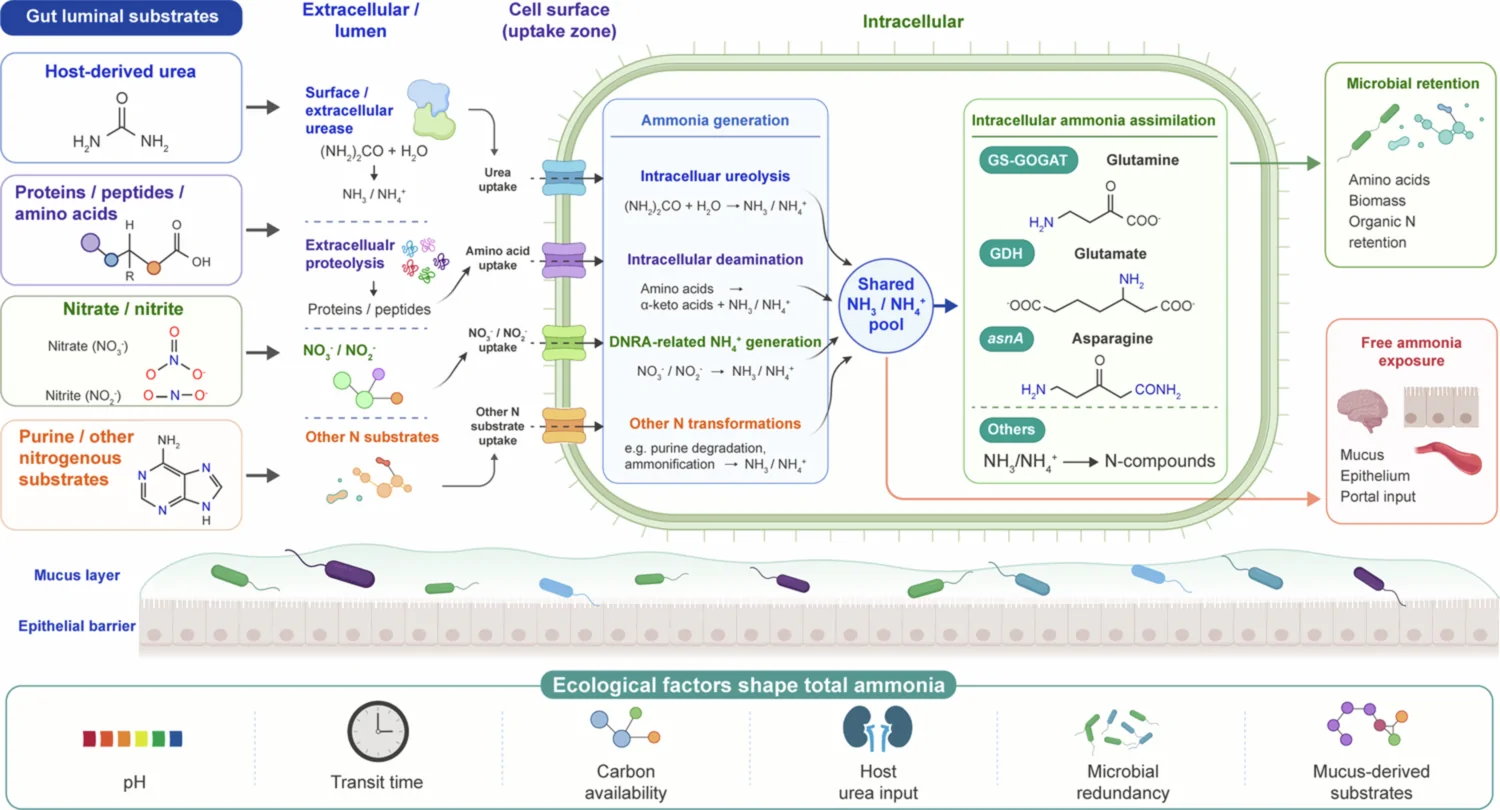
Cellular localization and ecological framework of gut microbial ammonia metabolism. This figure summarizes major microbial routes of ammonia production, assimilation, and retention in the gut with spatial resolution at the level of extracellular space, cell surface, and intracellular compartments. Ammonia is generated through multiple convergent pathways, including urease-mediated urea hydrolysis, extracellular proteolysis followed by intracellular amino acid deamination, DNRA-related nitrate/nitrite reduction, and other nitrogen substrate transformations. Urea hydrolysis may occur extracellularly or at the cell surface, or intracellularly after uptake. The resulting NH_3_/NH_4_^+^ enters a shared pool that may be assimilated via GS-GOGAT, GDH, asparagine synthetase-dependent incorporation, and related pathways, or retained in biomass and organic nitrogen through microbial retention and cross-feeding. When production exceeds assimilation capacity, free ammonia may accumulate and increase host exposure. The figure also highlights ecological factors that shift ammonia fate, including pH, transit time, carbon availability, host urea input, microbial redundancy, and mucus-derived substrates.

Three microbial reactions can supply ammonia to ileal or colonic luminal compartments (Figure 1). The first is urease-dependent urea hydrolysis. Because mammals do not express endogenous urease, urea delivered into the ileal or colonic lumen must be cleaved by microbial enzyme systems; consequently, urease-positive bacteria occupy a special position in host-derived urea nitrogen recycling and ammonia generation.^6^ ^,^ ^22^ This route is more likely to dominate from the ascending/proximal colon toward the distal colon when fermentable carbohydrate availability declines, transit time is prolonged, or host-derived protein substrates increase.

The second is proteolytic fermentation. Dietary proteins, peptides, and amino acids that are not fully absorbed in the small intestine, together with endogenous nitrogen-containing substrates derived from mucus, shed epithelium, and leaked proteins, can be continuously degraded and deaminated by microbes in the colon, thereby releasing ammonia and other related nitrogenous metabolites.^23^ ^,^ ^24^ This route is more likely to dominate from the ascending/proximal colon toward the distal colon when fermentable carbohydrate availability declines, transit time is prolonged, or host-derived protein substrates increase.

The third route is nitrate and nitrite reduction. Under anaerobic conditions, some bacteria can progressively reduce oxidized nitrogen and ultimately direct it toward ammonium production.^25^ ^,^ ^26^ These routes expand the biochemical sources of intestinal ammonium beyond urea hydrolysis and amino acid deamination. This pathway depends on the local availability of nitrate or nitrite and should not be generalized as a uniform process across all intestinal compartments. Together, these routes expand the biochemical sources of intestinal ammonium beyond urea hydrolysis and amino acid deamination.

These three routes correspond to different anatomical sites, ecological states, and substrate backgrounds. When host-derived urea is abundant and local pH or community structure favors urease-positive bacteria, urea hydrolysis can become prominent in ileal or colonic compartments exposed to urea delivery.^6^ ^,^ ^27^ When fermentable carbohydrates are reduced, transit time is prolonged, dietary protein is increased, or epithelial- and mucus-derived substrates increase, proteolysis and deamination are more likely to dominate, particularly in substrate-depleted distal colonic environments.^23^ ^,^ ^28^ ^,^ ^29^ Accordingly, the dominant ammonia-generating route depends on anatomical compartment, substrate delivery, transit time, pH, and the abundance of urease-positive or proteolytic taxa.

### 2.2 Urease-positive microbial communities and host nitrogen redistribution

Urease is a key microbial enzyme that catalyzes urea hydrolysis and releases ammonia, thereby redirecting nitrogen that the host would otherwise excrete as urea back into local intestinal nitrogen cycling.^1^ ^,^ ^6^ Because mammals do not express endogenous urease, this process depends almost entirely on urease-positive microorganisms.^6^ ^,^ ^18^ This process provides a direct biochemical link between host urea excretion and microbial nitrogen reuse.

Urea has long been regarded as a terminal excretory product after hepatic detoxification. However, isotope-tracing studies have shown that host-derived urea can be cleaved by bacterial urease, recaptured by the gut microbiota, and incorporated into amino acid synthesis and microbial biomass.^6^ Furthermore, colonization of treated mice with an engineered commensal *E. coli* expressing urease led to a shift of the gut microbiota toward Proteobacteria dominance and was accompanied by aggravation of immune-mediated colitis.^6^ These studies show that bacterial urease can cleave host-derived urea and incorporate the released nitrogen into microbial amino acid synthesis and biomass formation.

This transfer of host-derived urea nitrogen to microbial metabolism fits a broader vertebrate digestive pattern in which nitrogen substrates are delivered to the main fermentation site. Foregut fermenters such as ruminants deliver host-derived urea to the rumen for microbial ureolysis and microbial protein synthesis.^30^ ^,^ ^31^ Non-ruminant mammals rely more on distal small-intestinal, cecal, and ascending-colonic nitrogen recovery, where luminal microbes can hydrolyze urea, deaminate amino acids, and recycle ammonia into microbial biomass.^32^ In avian hindgut systems, nitrogenous substrates are handled closer to distal cecal or terminal gut compartments, consistent with the role of the avian ceca in microbial fermentation and nitrogen recycling.^33^ These comparative examples further support the view that nitrogen excretion can function as spatial substrate delivery to microbial fermentation sites rather than as uniform waste disposal.

Urease-positive bacteria therefore influence both luminal ammonia production and microbial access to host-derived urea nitrogen. Their downstream effects depend on several linked factors, including the amount of urea delivered to the gut, the abundance and activity of urease-positive taxa, the capacity of the community to assimilate ammonium, local pH, and the extent of epithelial or portal exposure. However, urease-positive bacteria should not be treated as a single functional group. They differ in how they access urea, where ureolysis occurs, and how the released nitrogen is used.

One important distinction is urea access. Urease-positive bacteria with dedicated urea channels or transport systems can import urea more efficiently and couple ureolysis to intracellular nitrogen assimilation and growth.^34^ ^,^ ^35^ Microbial strains that depend mainly on passive or facilitated urea entry are more dependent on local urea concentration and epithelial urea delivery. A second distinction is urease localization. Intracellular urease requires urea entry into the cell and may first support microbial nitrogen reuse before ammonia is released. In *Helicobacter pylori*, urease can also be recruited to the inner membrane together with UreI under acidic conditions, linking urea transport to local NH_3_/NH_4_^+^ handling.^36^ By contrast, surface-associated or extracellular urease can hydrolyze urea closer to the luminal or mucosal surface, increasing local NH_3_/NH_4_^+^ availability, altering pH, and potentially increasing epithelial exposure.^37^ ^,^ ^38^ These distinctions determine whether urea-derived nitrogen is primarily retained by urease-positive bacteria, shared with neighboring community members, or released into the lumen and portal-facing host interface.

### 2.3 Urease-independent microbial ammonia-generating programs

#### 2.3.1 Proteolysis-driven deamination

In addition to urea hydrolysis, proteolysis and deaminative fermentation constitute another major route of colonic ammonia generation, especially in ecological contexts characterized by high protein intake, low fermentable carbohydrate availability, prolonged transit time, or relative substrate depletion in the distal colon.^23^ ^,^ ^24^ ^,^ ^28^ Under these conditions, microbial biomass expansion may be limited by the carrying capacity of the local ecosystem, particularly when fermentable carbon sources are depleted. Nitrogen-containing substrates that exceed this assimilatory demand are then more likely to be degraded and deaminated, resulting in increased free-ammonia release. Early culture-based studies already suggested that *Bacteroides*, *Clostridial* groups, and members of *Enterobacteriaceae* play important roles in colonic protein degradation.^39^ In vitro fermentation studies with human fecal microbiota further showed that when protein and peptone were the sole fermentable substrates, *Enterobacteriaceae*, including *Escherichia-Shigella*, *Enterobacter*, and *Klebsiella*, as well as *Lachnospiraceae*, *Ruminococcaceae*, and some *Peptostreptococcaceae*-related colonies, expanded markedly; among them, *Lachnoclostridium*, *Paeniclostridium*, *Romboutsia*, *Dorea*, *Oscillibacter*, and *Anaerotruncus* were closely related to the accumulation of protein fermentation end products.^40^ These observations indicate a shift from carbohydrate fermentation to protein degradation and amino acid deamination. This metabolic state is often accompanied by a set of protein putrefaction-related end products. As carbohydrate-fermentation substrates decline, the microbiota relies more heavily on host- and diet-derived proteins for energy supply and growth. This process not only releases ammonia, but is also frequently accompanied by the accumulation of branched-chain fatty acids, amines, phenols, *p*-cresol, indole derivatives, and hydrogen sulfide.^23^ ^,^ ^24^ ^,^ ^40^ For example, *Clostridioides difficile* can generate *p*-cresol through decarboxylation of *p*-hydroxyphenylacetic acid and thereby gain a competitive advantage.^41^ ^,^ ^42^ Indole is commonly produced by *E. coli*, *Bacteroides* spp., and other tryptophan-metabolizing bacteria.^43^ ^,^ ^44^ H_2_S is closely associated with sulfur-reducing or sulfur-producing bacteria such as *Bilophila wadsworthia* and *Desulfovibrio* spp. and is more likely to increase under high-protein or high-sulfur-containing amino acid conditions.^45^ ^-^ ^47^ Together, these metabolites indicate increased protein fermentation in the distal colon. This state is often associated with higher luminal pH and accumulation of compounds such as ammonia, *p*-cresol, indole derivatives, and hydrogen sulfide, which can impair epithelial barrier function and promote local inflammation.

This state is especially likely to occur in the distal colon, because fermentable carbohydrates in this region are often consumed earlier by upstream communities, and prolonged residence time further amplifies substrate depletion.^23^ ^,^ ^28^ Concurrently, barrier injury, enhanced mucus degradation, and increased host-derived protein input can provide more substrate to proteolytic bacteria, further favoring deamination programs. Protein fermentation can involve several microbial groups. Some *Bacteroides* subgroups may act as primary proteolytic organisms that provide peptides and amino acids, whereas *Enterobacteriaceae*, *Lachnospiraceae*, *Ruminococcaceae*, and *Desulfovibrionaceae*, such as *Bilophila*, may further amplify the accumulation of ammonia, indole, *p*-cresol, and sulfides through subsequent fermentation and cross-feeding.^40^ Accordingly, when ammonia is observed to increase under certain disease or dietary patterns, the more important question is not solely whether urease is higher, but whether amino acid degradation and deamination are increased. Under these conditions, increased ammonia should be interpreted together with other proteolytic fermentation products, including branched-chain fatty acids, amines, indoles, *p*-cresol, and sulfides.

#### 2.3.2 Oxidized-nitrogen reduction and DNRA-driven ammonium generation

In addition to organic nitrogen cleavage, gut ammonia can also originate from the reduction of oxidized nitrogen. Under anaerobic conditions, some microorganisms can use dissimilatory nitrate reduction to ammonium (DNRA) to progressively reduce nitrate and nitrite and ultimately direct nitrogen toward ammonia generation.^48^ This process suggests that intestinal ammonia does not arise only from urea, proteins, and amino acids, but can also be generated through microbial transformation of oxidized nitrogen substrates. DNRA adds an oxidized nitrogen reduction route to the known microbial sources of intestinal ammonium.

Under experimental conditions approximating gastrointestinal oxygen tension, Tiso and Schechter demonstrated that after nitrate addition, *E. coli* and *Lactiplantibacillus plantarum* could both produce nitrite and ammonia.^26^ These findings indicate that nitrate and nitrite reduction can provide an ammonium-generating route for gut-associated bacteria when oxidized nitrogen substrates are locally available. The relevance of this pathway should not be described as uniform across the intestinal tract. Instead, it should be assigned according to local nitrate or nitrite availability, oxygen tension, redox state, inflammatory status, and microbial community structure. DNRA-related ammonium generation is therefore most likely to be relevant in sites such as the terminal ileum, cecal or ascending-colonic regions, distal anaerobic colonic compartments, and inflamed mucosal niches where host-derived nitrate availability may increase.^26^ Accordingly, DNRA complements organic nitrogen decomposition as a site- and condition-dependent source of intestinal ammonium.

#### 2.3.3 Additional microbial reactions that release ammonia

Beyond the two major routes described above, microbial ammonia generation in the gut has broader source heterogeneity. Different microbial groups can release ammonia through multiple reactions according to their metabolic capabilities, ecological niches, and available substrates. For example, clostridia such as *Clostridioides difficile* can perform Stickland-type amino acid fermentation, using amino acids such as proline as electron acceptors or donors; this represents an amino acid-centered program of deamination and nitrogen-flow redistribution.^49^ ^,^ ^50^ Meanwhile, *Clostridium sporogenes* and *E. coli* have been shown to carry out anaerobic purine degradation in the gut, suggesting that nucleotide and nitrogenous base metabolism can also contribute to local nitrogen cycling.^51^ In addition, under disease conditions, *Enterobacter cloacae* can increase fecal and circulating ammonium through dcd-related transformation of nitrogen-containing intermediates, indicating that non-urease, intermediate-metabolite-dependent ammonium-producing programs may also have important pathological significance.^52^ Together, these examples indicate that gut microbes can release ammonium through urease activity, amino acid fermentation, purine degradation, and dcd-related nitrogen transformations.

### 2.4 Microbial ammonia assimilation and nitrogen retention

If the source of ammonia is important, its fate is equally critical. Discussions have long focused mainly on ammonia-generating bacteria or high-ammonia exposure, while microbial recapture and fixation of ammonia have received less attention. Most bacteria possess the capacity to reincorporate free ammonia into central metabolic networks, commonly through glutamine synthetase-glutamate synthase pathway (GS-GOGAT), glutamate dehydrogenase (GDH), and other amidation-related programs. In organisms such as *E. coli* and *Corynebacterium glutamicum*, GS-GOGAT and GDH have been defined as two core ammonium assimilation pathways. *Bacillus subtilis* also uses GS-GOGAT as its main ammonium assimilation framework, and recent studies further show that under specific conditions it can reprogram nonclassical ammonium assimilation circuits.^53^ ^-^ ^55^ Through these pathways, free ammonia can be rapidly incorporated into glutamine, glutamate, and downstream nitrogen-containing metabolites and then enter microbial biomass. Accordingly, intestinal ammonia does not naturally remain for long periods as a free environmental molecule; its exposure level depends largely on whether the community has sufficient assimilation and fixation capacity.

Ecologically, ammonia assimilation reduces the amount of free ammonia available for epithelial absorption. In communities with strong assimilation capacity, much of the nitrogen produced as ammonia can be reabsorbed and retained as cellular biomass, amino acid reserves, or shared community resources, and thus may not produce substantial free-ammonia accumulation. In a chronic kidney disease-related study, human association analyzes linked *S. copri* with better kidney function status. In bacterial culture and engineered *E. coli* experiments, *asnA*-dependent ammonia assimilation reduced ammonia concentrations, whereas loss of *asnA* weakened this ammonia-lowering capacity 8. In animal models, *S. copri* or *asnA*-overexpressing bacteria alleviated ammonia-aggravated chronic kidney disease progression.^7^ Conversely, when assimilation capacity is weak, community function is disrupted, or cross-feeding or ammonium assimilation is reduced, the same amount of ammonia generation is more likely to produce local accumulation, increased epithelial exposure, and enhanced portal input. Comparative work by Reese et al. across the large intestines of multiple mammalian species further showed that the large intestine is a niche limited by total nitrogen supply; reducing dietary protein in mice markedly reduced fecal bacterial load, indicating that capture and retention of available nitrogen by the gut microbiota are themselves important constraints on community expansion.^10^ This finding indicates that once a community lacks sufficient nitrogen fixation and retention capacity, locally released ammonia is more difficult to refix and is more likely to appear as increased circulating ammonia.

### 2.5 Ammonia sharing and nitrogen allocation in cross-feeding networks

The large intestine is not necessarily a nitrogen-abundant environment, despite its high microbial density. Rather, available nitrogen can become limiting relative to the growth demand of dense microbial communities and the continuing input of carbon substrates. Reese et al., in a comparative study of the large intestines of 30 mammalian species, proposed that the large-intestinal microbiota generally occupies a niche limited by total nitrogen supply. They further showed in mice that reduced dietary protein decreases fecal bacterial load and observed stoichiometric gradients along the gut consistent with upstream host absorption.^10^ These findings indicate that colonic microbial growth can be constrained by the amount of nitrogen that remains available after upstream host absorption.

In this context, free ammonia, especially NH_4_^+^, is better regarded as an exchangeable nitrogen source that can be competed for, shared, and reused. In a functional genetic study of *Bacteroides thetaiotaomicron*, Liu et al. found that this bacterium can adjust the synthesis of nitrogen-containing precursors according to ammonium availability and use alternative enzyme systems for biosynthesis of arginine, lysine, and other compounds. These observations indicate that NH_4_^+^ is an actively sensed and utilized nitrogen source for gut commensals.^56^ Doranga et al. further demonstrated that *E. coli* can use multiple nitrogen sources, including ammonia, to colonize the mammalian intestine.^57^ In addition, colonic NH_4_^+^ is produced by microbes and increases distally; growth of *Bacteroides* spp. can depend on NH_4_^+^; and ammonium released by urease-positive bacteria can be used by nonurease bacteria.^58^ These studies indicate that ammonium produced by one group of microbes can become a nitrogen source for other community members.

Nitrogen cross-feeding provides a mechanism by which ammonia released from urea hydrolysis, amino acid deamination, or other nitrogen-transforming reactions is redistributed within the microbial community. Ammonia producers release nitrogen into the environment through urea hydrolysis or deamination programs; assimilators rapidly fix this nitrogen through GS-GOGAT, GDH, and other amidation pathways; and other members further reuse it through amino acids and related metabolites. Pérez Escriva et al. systematically mapped nitrogen and carbon cross-feeding networks in a 10-member synthetic mouse gut community and showed that nitrogen exchange is not a marginal phenomenon, but nitrogen exchange occurred together with carbon exchange in this defined community.^59^ This study provides direct evidence that nitrogen released by one member can be captured and reused by others in a defined gut microbial community.

Nitrogen cross-feeding therefore determines how rapidly ammonium released by one taxon is incorporated by other community members into amino acids, nucleotides, or biomass. When cross-feeding and assimilatory capacity are intact, free ammonia is more likely to be retained within microbial metabolic networks. When these capacities are reduced, more free ammonia may remain in the lumen and become available for epithelial or portal exposure.^10^ ^,^ ^58^ ^,^ ^59^

### 2.6 Ammonia speciation and spatial heterogeneity

The pKa of ammonia is approximately 9.15. At physiological pH 7.4, the vast majority exists as NH_4_^+ 1^. Accordingly, small local changes in pH may not substantially alter measured total ammonia, but can be sufficient to change ammonia speciation, membrane diffusibility, and the form of local exposure. In comparison, NH_3_ more readily diffuses across membranes, whereas NH_4_^+^ is more constrained by ion channels, transporters, and the local ionic environment.^1^ The distal colonic epithelium does not have equivalent permeability to NH_3_ and NH_4_^+^, and luminal HCO_3_^-^ secretion and local acid–base status can markedly regulate total NH_4_^+^ absorption.^60^ ^-^ ^62^ Accordingly, total ammonia should be reported with pH because pH changes the NH_3_/NH_4_^+^ ratio and membrane permeability: what determines host exposure is not total ammonia itself, but how much of it exists in diffusible, transportable, and damaging forms.

Ammonia also contributes to the local acid-base environment of the colon. Acidification of the proximal colon is primarily driven by short-chain fatty acids generated through fermentation of microbially accessible carbohydrates. Moving distally along the colon, as short-chain fatty acids are absorbed, epithelial bicarbonate secretion increases, and proteolytic metabolism and ammonia release increase, luminal pH gradually rises. Together, these processes gradually raise luminal pH. After depletion of microbially accessible carbohydrates, the metabolic transition from carbohydrate fermentation to proteolysis and the accompanying ammonia release constitute an important mechanism of distal colonic alkalinization.^27^ Thus, ammonia is not only affected by luminal pH; ammonia production can also help shape pH, especially in the distal colon.

The role of pH is not limited to chemical gating, it also reshapes microbial metabolic modes and community selection in the reverse direction. Lower colonic pH usually favors carbohydrate fermentation and related microbes, whereas higher pH is more often associated with proteolysis, increased branched-chain fatty acids, and protein putrefaction metabolism.^27^ Roager et al. further showed that longer colonic transit time is accompanied by higher distal pH, more proteolytic features, and changes in metabolic end products.^28^ This finding indicates that pH both determines the form in which ammonia exists and influences which ammonia-producing ecology the community is more likely to enter.

Anatomical location determines both the chemical form of ammonia and the tissue surface exposed. The ileum, ascending or proximal colon, distal colon, mucus layer, luminal center, and feces should be treated as distinct compartments rather than as interchangeable intestinal samples. The ileum combines host secretion, rapid absorption, epithelial glutamine metabolism, host-derived urea delivery, and direct portal drainage. Therefore, ammonia generated or absorbed in the ileal compartment may rapidly enter host metabolic handling. The ascending or proximal colon contains a denser microbial community and is a major site where residual dietary substrates, host-derived urea, microbial fermentation, urea hydrolysis, ammonia assimilation, and nitrogen cross-feeding intersect. The distal colon and feces more strongly reflect terminal fermentation, longer residence time, water removal, reduced fermentable carbohydrate availability, and proteolysis-associated alkalinization.^10^ ^,^ ^27^ ^,^ ^63^

Detecting elevated ammonia alone is therefore insufficient to define its source, exposure route, or biological consequence. The key is where ammonia is measured, in what chemical form it exists, and whether the final exposure involves the ileal epithelium, the ascending-colonic fermentation compartment, the distal-colonic or fecal terminal state, portal input, or systemic circulation. Portal ammonia more closely reflects gut-derived input, fecal ammonia is closer to the terminal state of the distal colon, and peripheral blood ammonia is the residual value after hepatic and extrahepatic buffering. These three are not necessarily linearly correlated and cannot substitute for one another.^1^ Accordingly, pH and spatial localization are not accessory information, but necessary conditions for interpreting the ammonia exposure. Only by placing ammonia back into its specific local environment and spatial hierarchy can we determine whether ammonium is incorporated into microbial biomass or remains available for epithelial, portal, or systemic exposure.

## 3 Layered host sensing and organ responses to microbiota-derived ammonia

**Figure 2.**
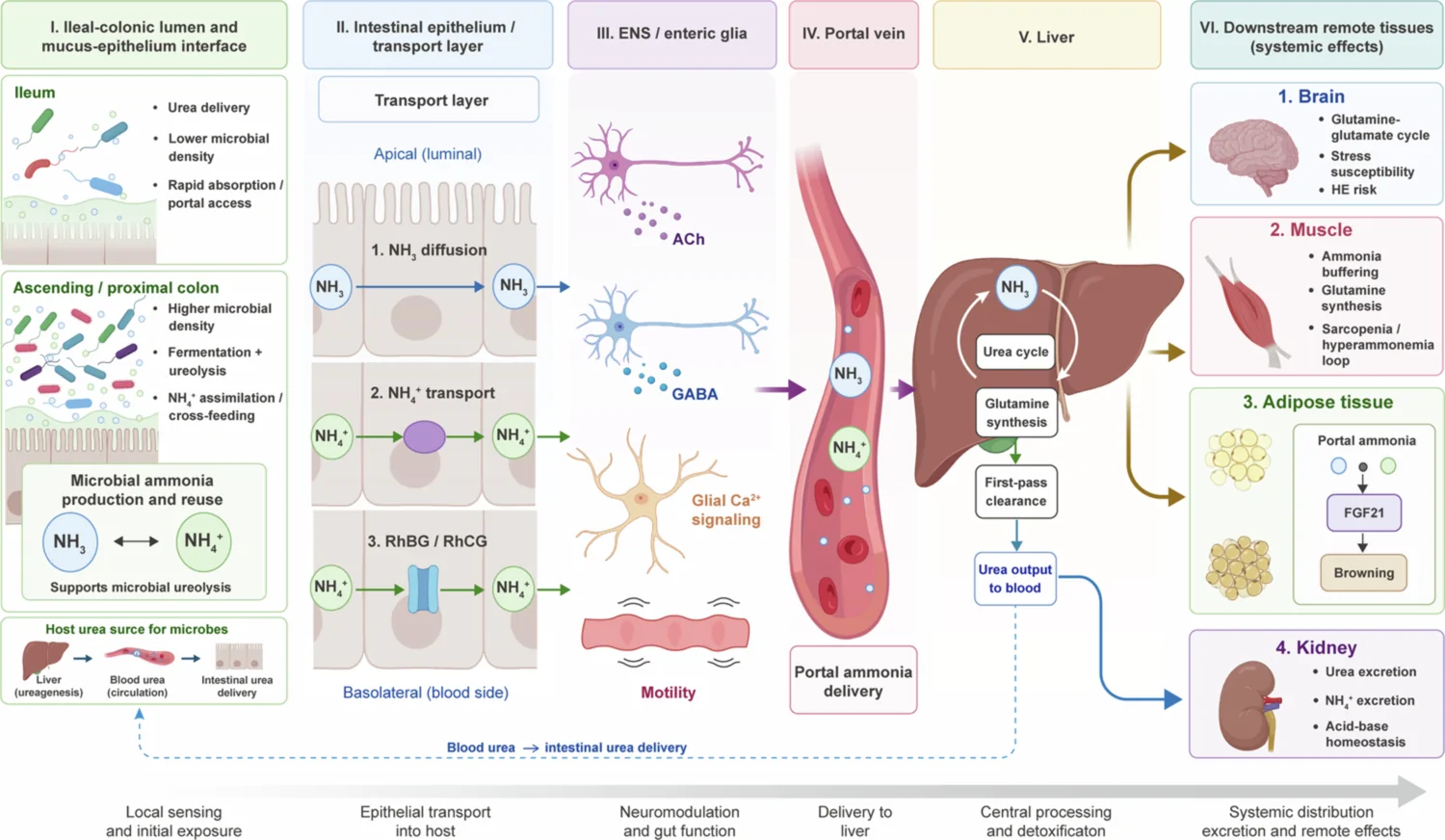
Host interfaces that reshape microbiota-derived ammonia across intestinal, portal, hepatic, renal, and systemic compartments. This figure illustrates how microbiota-derived ammonia is transported, metabolized, redistributed, and excreted across host interfaces. The intestinal compartment is separated into the ileum and ascending/proximal colon. The ileum is characterized by urea delivery, lower microbial density, rapid absorption, and direct portal access, whereas the ascending/proximal colon has higher microbial density and supports fermentation, ureolysis, ammonium assimilation, and nitrogen cross-feeding. Host-derived urea generated through hepatic ureagenesis enters the circulation and can either return to the intestinal lumen, providing substrate for microbial ureolysis and nitrogen recycling, or undergo renal excretion. Locally generated NH_3_/NH_4_^+^ crosses the mucus-epithelial interface through pH-dependent speciation and polarized epithelial transport, enters the portal circulation, and is processed by the liver through ureagenesis, glutamine synthesis, and first-pass clearance. The kidney further contributes to systemic nitrogen handling through urea excretion, NH_4_^+^ excretion, and acid–base homeostasis. When hepatic clearance is impaired or portosystemic shunting is present, ammonia may spill over into the systemic circulation and affect distal organs, including the brain, skeletal muscle, adipose tissue, and enteric nervous system.

Microbiota-derived ammonia metabolism begins to affect the host at the local intestinal interface and then gradually extends to extraintestinal tissues through trans-barrier transport, portal input, and hepatic integration. Current studies indicate that the physiological significance of ammonia presence is not determined by a single concentration change, but by its source, chemical form, exposure site, tissue-processing capacity, and host state (Figure 2).

### 3.1 Ileal and colonic epithelial-mucosal interfaces as initial host exposure sites

The ileal and colonic epithelial-mucosal interfaces are major sites where microbiota-derived or microbiota-modified ammonia can reach host tissues.^64^ ^,^ ^65^ These interfaces differ in microbial density, transit time, urea delivery, pH, mucus structure, epithelial transport, and portal drainage.^60^ ^,^ ^66^ Therefore, ileal and colonic ammonia exposure should not be treated as a single intestinal process.

**Figure 3.**
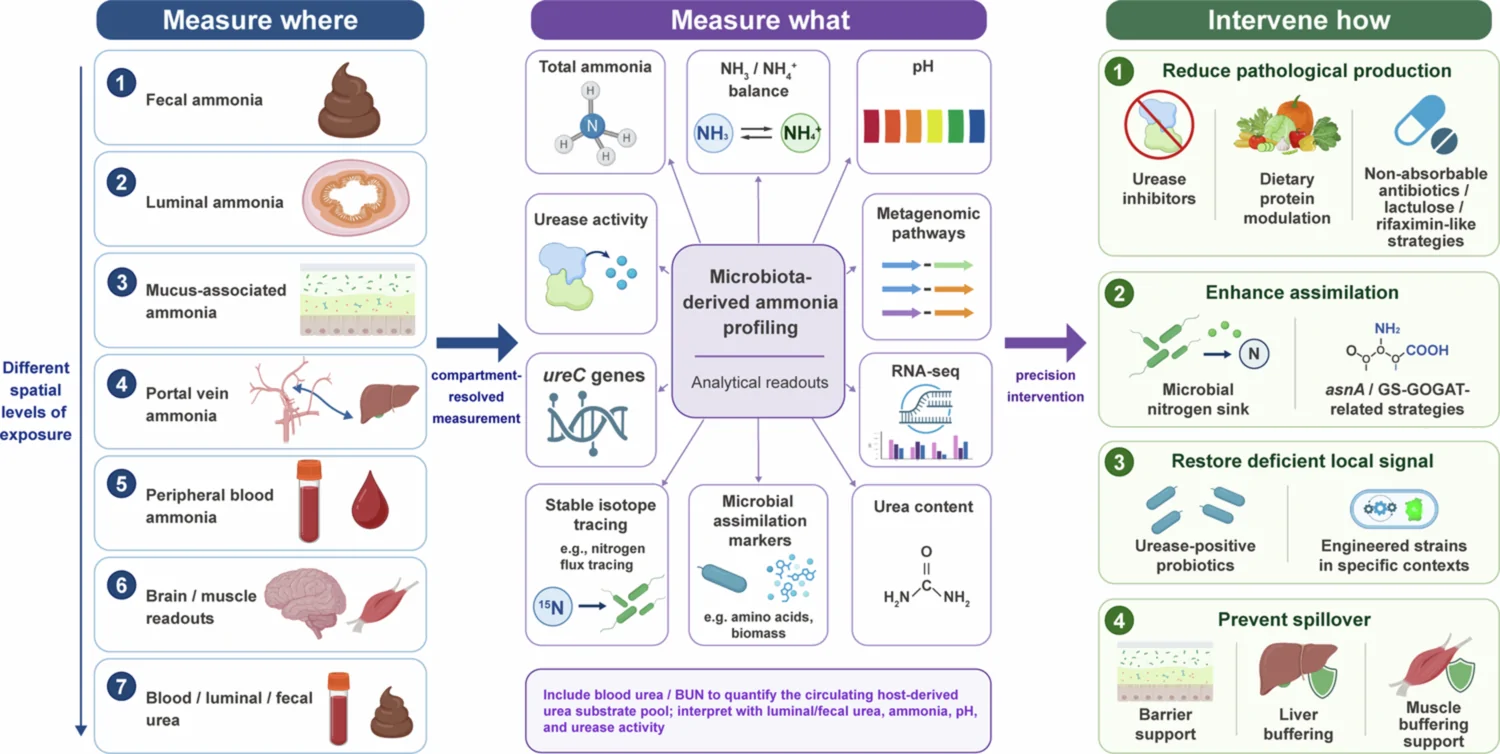
Measurement and intervention framework for microbiota-derived ammonia. This figure outlines a compartment-resolved framework for evaluating and targeting microbiota-derived ammonia. Relevant measurement sites include fecal, luminal, mucus-associated, portal, peripheral blood, and distal tissue readouts. Blood, luminal, and fecal urea should also be assessed because they reflect the host-derived urea substrate pool available for microbial ureolysis, although they are not direct ammonia-exposure readouts. Key analytical dimensions include total ammonia, NH_3_/NH_4_^+^ balance, pH, urease activity, ureC genes, metagenomic or RNA-seq signatures, microbial assimilation markers, stable isotope tracing, and urea content. These measurements should be interpreted together with host vulnerability factors, including barrier integrity, liver function, portosystemic shunting, renal handling, muscle buffering, and neurological susceptibility. Intervention strategies should be matched to context, including reducing pathological production, enhancing microbial assimilation, restoring deficient local signals, or preventing ammonia spillover through barrier, liver, and muscle support.

Classical human colonic studies have shown that the colon is both an important site of bacterial ammonia generation and an active site where urea entering the lumen is decomposed again and converted to ammonia; thus, the intestinal epithelium is continuously exposed to microbial ammonia from the outset.^65^ ^,^ ^67^ Furthermore, human excluded-colon perfusion experiments and in vivo human colonic ^15^ *N*-urea metabolic studies have demonstrated that the colonic mucosa directly participates in urea/ammonia transmucosal transport and local nitrogen cycling. This is echoed by experiments in which colonic luminal ammonium perfusion in pigs increased portal glutamine and arginine, indicating that locally generated or introduced ammonia in the gut can rapidly enter host metabolic pathways.^64^ ^,^ ^68^ ^,^ ^69^ Together, these human and animal perfusion studies support the intestinal epithelial-mucosal interface as a key anatomical site where luminal ammonia is converted into portal metabolic exposure.

Urease-positive *Streptococcus thermophilus* and *Streptococcus salivarius* should be interpreted as microbial physiology examples of commensal urease activity rather than as major general contributors to colonic ammonia metabolism. *Helicobacter pylori* provides a separate gastric mucosal pathogen model in which bacterial urease-derived ammonia can affect epithelial junctions under a specific pathological niche. These examples illustrate that urease-derived ammonia can influence mucosal physiology, but the anatomical niche and microbial source must be specified when interpreting their relevance to intestinal ammonia exposure.

In vitro epithelial cell culture studies show that ammonia can alter oxidative stress, mitochondrial function, and tight-junction integrity. Ammonia can damage the Caco-2 tight junction barrier by inducing oxidative stress and is accompanied by mitochondrial dysfunction.^70^ Even within feces-related concentration ranges, ammonia itself is sufficient to increase paracellular permeability in Caco-2 monolayers.^71^ In *H. pylori*-related epithelial models, bacterial ammonia production induced abnormal occludin processing and disrupted tight junctions.^72^ In animal models of intestinal ammonia exposure, ammonia was associated with epithelial ROS accumulation, apoptosis, reduced barrier-protein expression, villus shortening, inflammatory infiltration, and junctional disruption.^73^ ^,^ ^74^ These cell culture and animal studies indicate that ammonia exposure can impair epithelial barrier function, although the specific response depends on the model system and exposure conditions.

Collectively, evidence from human colonic studies, pig perfusion experiments, in vitro epithelial models, gastric pathogen models, and animal intestinal injury models supports the intestinal epithelial-mucosal interface as an initial site of host exposure to microbiota-derived ammonia. At this interface, ammonia may remain locally buffered, cross the epithelial barrier, enter the portal circulation, or contribute to epithelial stress and barrier dysfunction.^64^ ^,^ ^68^ ^,^ ^70^

### 3.2 Microbiota-derived ammonia shapes enteric neural and neurotransmitter signaling networks

The enteric nervous system is a local tissue system that can respond to ammonia and may mediate changes in intestinal motility and neurotransmitter signaling.^75^ ^,^ ^76^ Mouse dysmotility models and related human constipation samples provide model-specific evidence that local ammonia production is associated with altered colonic acetylcholine signaling and motility regulation.^75^ ^,^ ^77^ In this section, we focus on host-side neural mechanisms that may connect ammonia exposure with enteric neuronal and glial responses.

Evidence from ex vivo and in vivo experimental systems further supports a connection between ammonia exposure and enteric neural activity. In ex vivo small-intestinal neuromuscular preparations, Fried et al. found that ammonia altered neuromuscular transmission and proposed that myenteric plexus glial cells may translate local ammonia exposure into altered neural output through *γ*-aminobutyric acid (GABA) release.^76^ In an in vivo pig ammonia-exposure model from birth, sustained ammonia exposure altered the number and area of ileal enteric neurons and glial cells and increased choline acetyltransferase (ChAT) and galanin expression.^78^ These studies indicate that ammonia can affect enteric neuromuscular signaling and ENS plasticity in experimental models, although the dose range and physiological relevance need to be defined in each setting.

The cholinergic pathway provides a plausible mechanism linking local ammonia exposure to motility regulation. In a mouse genetic model, developmental deletion of enteric ChAT caused gastrointestinal dysmotility, slowed colonic transit, and dysbiosis, indicating that cholinergic transmission is a key determinant of intestinal motility phenotypes.^77^ In enteric glial preparations and ex vivo motility studies, cholinergic activation of enteric glia generated Ca^2+^ responses and regulated gastrointestinal motility.^79^ ^-^ ^81^ These findings support the idea that changes in acetylcholine availability or transmitter environment can be transmitted through enteric glia to affect network-level motility responses.

Additional evidence comes from studies of GABA signaling and enteric neuronal excitability. In ex vivo myenteric plexus preparations and cultured myenteric neurons, GABA transporters, depolarization-evoked GABA release, GABA-mediated regulation of neuronal excitability, GABA-induced Ca^2+^ signaling, and receptor-induced Ca^2+^ responses have been documented.^82^ ^-^ ^85^ signaling pathway, but direct evidence linking microbiota-derived ammonia to this pathway remains limited to selected experimental models.

Together, current evidence suggests that microbiota-derived ammonia can influence enteric neuron-glia-transmitter circuits in specific experimental contexts. The strongest causal evidence comes from mouse dysmotility intervention models, whereas human data are currently mainly associative and derived from related constipation samples. Therefore, the ammonia-enteric neurotransmitter axis should be framed as a model-supported mechanism that requires further validation in human physiological and disease settings.

### 3.3 Trans-barrier ammonia transport determines host exposure patterns

Actual host exposure to microbiota-derived ammonia is first determined by ammonia speciation and trans-barrier transport, rather than by total luminal ammonia alone. Unprotonated NH_3_ more readily diffuses across membranes, whereas NH_4_^+^ is more constrained by local pH, ionic gradients, and transport processes. Therefore, total ammonia concentration does not define epithelial exposure unless pH, NH_3_/NH_4_^+^ speciation, apical versus basolateral transport, and barrier integrity are also considered. In early colonic crypt micro-perfusion studies, the apical membrane of colonic crypts showed much lower permeability to NH_3_/NH_4_^+^ than the basolateral membrane, and intracellular pH measurements showed different cellular entry kinetics for NH_3_/NH_4_^+ 60, 62^. Earlier work also indicated that luminal pH can substantially influence ammonia transport and that NH_4_^+^/H^+^ exchange can occur at the brush border of the small intestine.^86^ ^-^ ^88^ These findings indicate that ileal and colonic ammonia transport cannot be explained by passive NH_3_ diffusion alone.

This differentiated exposure is further supported at the molecular level. In mouse gastrointestinal tissue, Rhesus blood group B glycoprotein (RhBG) and Rhesus blood group C glycoprotein (RhCG) are expressed in a polarized epithelial pattern, with RhCG enriched on the apical side facing the lumen and RhBG enriched on the basolateral side facing the interstitium and blood supply.^66^ ^,^ ^89^ Here, polarized NH_4_^+^ transport means that ammonium movement is not uniform across the epithelial cell layer: the luminal-facing and tissue-facing sides of epithelial cells have different transport proteins, ionic gradients, and chemical environments. This allows NH_4_^+^ movement to differ depending on whether ammonia/ammonium is presented from the luminal side or the basolateral side. Human excluded-colon perfusion studies and human small-intestinal transport studies show that colonic and small-intestinal epithelia can both absorb and secrete ammonia, and that transepithelial movement cannot be explained by passive diffusion alone.^64^ ^,^ ^65^ ^,^ ^86^ In epithelial ion-transport experiments, apical and basolateral ammonia exposure produced different effects on Cl^-^ secretion.^90^ Thus, the same total ammonia concentration can produce different epithelial responses depending on where ammonia is present and which side of the epithelial layer is exposed.

This spatial difference also extends from local epithelial exposure to portal and systemic circulation. In intact pig colonic perfusion experiments, luminal ammonium salts increased portal ammonia, glutamine, and arginine, whereas lower doses did not synchronously raise arterial ammonia.^65^ ^,^ ^67^ ^,^ ^68^ These observations suggest that transepithelial transport first shapes local and portal exposure before producing systemic changes. Mucus-layer status, epithelial integrity, and local metabolic activity further influence the distribution and accessibility of ammonia. Subsequent animal and epithelial transport studies also suggest that gastrointestinal ammonia absorption is not always dependent on a simple NH_3_ gradient. In some models, even when intestinal tissues express Rh proteins, ammonia absorption can show relative independence from chemical gradients; in avian colon studies, active electrogenic NH_4_^+^ secretion has also been observed, indicating that intestinal ammonia handling can include active secretory processes.^91^ ^,^ ^92^

Together, these studies indicate that host exposure to microbiota-derived ammonia should be defined by chemical form, pH, apical and basolateral transport, epithelial barrier status, and whether absorbed ammonia enters portal or systemic circulation. The key experimental question is therefore not only how much total ammonia is present, but which form of ammonia is present, where it is located, and whether it reaches the epithelium, portal vein, or systemic circulation.^60^ ^,^ ^68^

### 3.4 The liver as the primary extraintestinal integrator of microbiota-derived ammonia

The liver is the first and most critical extraintestinal organ encountered by microbiota-derived ammonia after it enters the portal circulation.^93^ ^-^ ^95^ For gut-derived ammonia, the liver does not perform only unidirectional clearance; it extracts ammonia from portal blood, converts some nitrogen into urea or glutamine, and limits entry into systemic blood.^94^ ^,^ ^95^ Early perfused-liver and hepatic lobular zonation studies showed that hepatic ammonia handling has clear spatial division of labor: the periportal zone tends toward urea synthesis, whereas the perivenous zone tends toward glutamine resynthesis, thereby forming efficient clearance and compensatory assimilation of portal ammonia.^95^ ^-^ ^97^ These observations indicate that before gut-derived ammonia leaves the portal level, the liver determines whether it is rapidly cleared, reassimilated, or allowed to spill over.

This function has subsequently been directly quantified. A porcine positron emission tomography (PET) model measured hepatic ammonia metabolism and further showed that hepatic microcirculatory architecture and perfusion patterns markedly affect clearance efficiency.^93^ ^,^ ^94^ This finding indicates that the liver is not only a metabolic buffer, but also a key limiting link that determines whether a local intestinal signal can become systemic exposure. Microbial intervention studies further show that the amount of ammonia reaching the liver changes with microbial urea hydrolysis and deamination. In mouse models, engineered low-urease microbial communities reduced intestinal urea degradation and systemic hyperammonemia and improved liver failure-related outcomes. In complex-microbiota mouse models, the bacterial urease inhibitor benurestat suppressed host ammonia burden.^8^ ^,^ ^22^ These studies connect microbial ammonia production, portal ammonia input, and hepatic first-pass processing in defined animal models.

Disease-related evidence further shows that impaired hepatic handling can convert microbial ammonia input into systemic metabolic burden. In mouse models and human samples of alcohol-associated fatty liver disease, gut-derived ammonia has been linked to ethanol metabolism and activating transcription factor 4 (ATF4)-dependent lipogenesis. In NAFLD-related human and experimental studies, urea-cycle dysregulation and ammonia accumulation have been incorporated into disease-mechanism frameworks.^98^ ^,^ ^99^ In clinical studies of patients with cirrhosis complicated by Helicobacter pylori infection, blood ammonia was increased in some patients, and eradication therapy reduced hyperammonemia in part of the clinical population.^100^ ^,^ ^101^ These findings suggest that when hepatic detoxification capacity is impaired, additional microbial or gastric urease-derived ammonia input is more likely to appear as detectable systemic hyperammonemia.

Together, evidence from experimental liver models, porcine in vivo imaging, mouse microbiota-intervention models, and human liver-disease studies supports the liver as a central first-pass organ for gut-derived ammonia. Through zonated metabolism, portal extraction, and impaired handling under liver disease conditions, the liver determines whether microbiota-derived ammonia remains mainly at the portal level, is reassimilated into host nitrogen metabolism, or spills over to produce systemic organ exposure.^94^ ^,^ ^98^

### 3.5 Secondary responses of distal organs to microbiota-derived ammonia

Responses of distal organs to microbiota-derived ammonia differ by organ system and by evidence type. In liver disease and systemic hyperammonemia, elevated ammonia has strong clinical and mechanistic support as a pathological factor, particularly in the brain and skeletal muscle. In contrast, evidence that microbiota-derived ammonia contributes to physiological or adaptive distal-organ responses is more limited and comes mainly from selected animal models. Therefore, extraintestinal effects should be interpreted according to the site of exposure, the level of systemic ammonia, liver function, and the experimental system in which the evidence was obtained.

The brain provides the clearest example of this evidence asymmetry. In human hepatic encephalopathy and experimental hyperammonemia models, ammonia-induced astrocyte swelling, disruption of the glutamate–glutamine cycle, oxidative stress, and mitochondrial dysfunction are well-established mechanisms of neurological injury.^4^ ^,^ ^102-105^ Astrocytic glutamine synthetase is central to brain ammonia handling and glutamate‒glutamine cycling, and excessive ammonia exposure can drive intracellular glutamine accumulation and osmotic stress.^4^ ^,^ ^105^ By contrast, evidence that reduced microbiota-derived ammonia can impair brain glutamine availability is currently based mainly on selected mouse models.^9^ This evidence is consistent with a model-specific link between microbial urease activity, ammonia-derived nitrogen input, and brain glutamine metabolism, but it should not be interpreted as evidence that low ammonia is a general cause of neurological disease.

Skeletal muscle represents another key secondary response organ because it is both an important peripheral ammonia buffer and a direct target of high-ammonia exposure. In mouse skeletal muscle, RhBG/RhCG transporters are expressed, suggesting a transporter-associated basis for muscle ammonia handling.^106^ In hyperammonemic rodent models of cirrhosis, ammonia induces muscle autophagy, increases myostatin transcription through an NF-kB-dependent pathway, impairs mitochondrial respiration, enhances oxidative stress, and promotes metabolic changes resembling amino acid deprivation.^107^ ^,^ ^108^ In cirrhosis-related hyperammonemic animal models, ammonia-lowering treatment reversed muscle phenotypes and restored proteostasis-related molecular abnormalities.^109^ ^,^ ^110^ Compared with the brain, current muscle evidence mainly supports ammonia as a pathological burden under high-exposure conditions rather than as an adaptive microbial signal.

Microbiota-derived ammonia has also been linked to metabolic adaptation under protein restriction, but this evidence is model-specific. In mouse protein-restriction models, gut microbiota depletion weakens hepatic fibroblast growth factor 21 (FGF21) induction, energy metabolic remodeling, and white-adipose browning.^111^ ^-^ ^113^ Defined-community mouse models further suggest that microbial nitrogen metabolism can increase portal ammonia and contribute to hepatic FGF21 induction under low-protein dietary conditions.^114^ These findings indicate that microbiota-derived ammonia may participate in host metabolic adaptation under defined nutritional conditions. However, this pathway should be framed as a low-protein diet-specific and model-supported mechanism rather than a general function of gut-derived ammonia.

Overall, distal-organ responses should be separated into established pathological effects of elevated systemic ammonia and more limited, model-specific evidence for adaptive ammonia-related signaling. The strongest evidence supports ammonia-mediated injury in human hepatic encephalopathy and experimental hyperammonemia models, whereas the roles of microbiota-derived ammonia in brain glutamine supply, FGF21 induction, and adipose remodeling require further validation across additional animal models and human studies.

### 3.6 From local exposure to systemic phenotypes

The biological consequence of microbiota-derived ammonia depends first on the spatial layer at which it rises, not simply on whether it rises. Early comparisons between germ-free and conventionally raised guinea pigs showed that portal and peripheral blood ammonia are not equivalent, and that the presence of the microbiota alters this gradient relationship.^115^ Recent multi-site venous sampling in patients with cirrhosis further showed significant differences in ammonia within the splenic, mesenteric, and portal venous systems themselves. For example, mesenteric venous ammonia is higher than splenic venous ammonia, whereas portal venous ammonia distribution becomes relatively homogeneous after entering the liver.^116^ These observations indicate that portal ammonia is not a single uniform variable, but an exposure compartment with internal spatial structure.

Blood ammonia levels are also strongly influenced by blood flow routes and the degree of shunting, not solely by changes in intestinal production. In early human clinical studies, ammonia tolerance testing was used as an indirect index of portosystemic collateral circulation. Later clinical studies of spontaneous collateral circulation and intrahepatic shunting further showed that bypass of hepatic first-pass clearance strongly modifies systemic ammonia exposure.^117^ ^-^ ^119^ This also explains why, in clinically stable patients with cirrhosis, venous ammonia cannot be simply equated with grading of neurological symptoms but can still predict hospitalization and mortality, because it partially integrates the combined results of intestinal production, portosystemic shunting, hepatic processing, and peripheral clearance.^120^ ^,^ ^121^

Human portal-venous sampling studies and experimental hepatic zonation studies together show that local intestinal exposure, portal exposure, and peripheral circulating exposure are not equivalent ammonia compartments. This distinction is especially important for ammonia because its systemic availability is shaped by hepatic lobular detoxification, portal blood flow direction, epithelial barrier state, and distal-organ susceptibility. Therefore, the final phenotype associated with microbiota-derived ammonia should be interpreted according to its source, chemical form, local barrier state, portal input, hepatic first-pass capacity, and distal-organ vulnerability.

## 4 Physiological adaptation and disease burden in host responses to microbial ammonia metabolism

Ammonia-related outcomes differ substantially across physiological and disease settings, depending on ammonia load, anatomical exposure compartment, and host clearance capacity. In liver disease and systemic hyperammonemia, elevated peripheral ammonia has strong clinical and mechanistic support as a pathological factor. In contrast, evidence for local ammonia as part of compensatory or adaptive responses is more limited and is mainly derived from selected animal models, nutritional adaptation studies, and defined microbial community experiments. Table 1 summarizes these contexts by ammonia source, exposure compartment, host condition, and evidence type.

### 4.1 From local input to systemic burden

Under normal physiological conditions, continuous ammonia generation by the gut microbiota does not necessarily lead to systemic injury, because locally generated ammonia can be restricted, metabolized, and redistributed at levels such as the intestinal epithelium, portal vein, and liver.^64^ ^,^ ^94^ Systemic hyperammonemic burden usually forms through the superposition of two processes: abnormal enhancement of microbial ammonia-generating programs and simultaneous decline in the host's ability to restrict and handle portal nitrogen flow.^94^ ^,^ ^122^

Human cirrhosis cohorts provide the clearest clinical evidence linking microbial ammonia generation to systemic ammonia burden. In patients with cirrhosis, intestinal or fecal microbial urease activity correlates with plasma ammonia levels, and elevated stool urease activity predicts overt hepatic encephalopathy.^123^ Human microbiome studies further showed enrichment of *Proteobacteria*, *Fusobacteria*, *Streptococcaceae*, and *Veillonellaceae* in patients with cirrhosis compared with healthy controls.^124^ In human decompensated cirrhosis and hepatic encephalopathy cohorts, urease-positive bacteria were enriched in the hepatic encephalopathy group and were associated with elevated blood ammonia and brain glutamine.^125^ In human metagenomic studies of cirrhosis, fecal strains also showed overlap with oral-derived strains, suggesting possible oral-to-gut microbial translocation; however, this should be interpreted as a supportive association rather than definitive evidence that oral urease-producing bacteria generally drive hyperammonemia.^126^

The ammonia-generating microbial shift in liver disease is not limited to urease-positive taxa. In human cirrhosis and hepatic encephalopathy cohorts, *Enterobacteriaceae*, *Veillonella*, *Streptococcus*, *Enterococcus*, and other taxa associated with proteolytic fermentation are increased, whereas indigenous commensals related to short-chain fatty acid production and nitrogen fixation are reduced.^127^ ^,^ ^128^ These changes indicate a shift toward microbial pathways that favor proteolysis, deamination, and free ammonia release. In experimental mouse models, small-molecule gut bacterial urease inhibitors reduced intestinal bacterial urease activity, lowered hyperammonemic burden, and improved liver disease-related outcomes.^22^ Together, human cohort data and mouse intervention studies support gut microbial ammonia generation as an upstream contributor to systemic hyperammonemia in liver disease.

Host-side defects determine whether locally generated ammonia becomes systemic exposure. In patients with cirrhosis, reduced gastric acid secretion, impaired small-intestinal motility, altered bile acid profiles, weakened antimicrobial defense, and intestinal barrier injury promote bacterial overgrowth and trans-barrier movement of microbial products.^129^ ^,^ ^130^ Clinical studies further show that hepatic ammonia extraction is associated with intrahepatic portosystemic shunting, and blood ammonia independently predicts liver-related hospitalization and death in stable cirrhosis.^117^ ^,^ ^120^ These findings indicate that systemic hyperammonemia in cirrhosis reflects both increased gut-derived ammonia input and impaired hepatic first-pass handling.

These microbial changes indicate increased protein degradation and amino acid deamination, which can release more free ammonia into the colonic lumen. Urease activity changes in the same direction as blood ammonia,^123^ and enrichment of urease-positive bacteria and their urea-degrading capacity are also associated with hepatic encephalopathy phenotypes.^125^ Existing evidence clearly suggests that in liver disease, the intestinal microbial nitrogen metabolic network as a whole shift toward ammonia-generating programs. Intervention studies further support microbial metabolism as an important upstream intervention point for hyperammonemic burden. Small-molecule gut bacterial urease inhibitors have been shown in experimental models to inhibit intestinal bacterial urease, reduce hyperammonemic burden, and improve liver disease-related outcomes. These observations indicate that reducing hyperammonemic burden can be approached not only from the host clearance side, but also from the microbial ammonia-production side in the gut.

Elevated microbial ammonia can also contribute to systemic phenotypes beyond classical hepatic encephalopathy, but the evidence differs by disease setting and model system. In human multiple myeloma cohorts, Enterobacter cloacae was enriched in patients with osteolysis. In mouse models and osteoclast-related experimental systems, E. cloacae promoted osteoclastogenesis and osteolysis by increasing circulating ammonium levels.^52^ In mouse models and human samples of alcohol-associated fatty liver disease, chronic alcohol exposure was associated with elevated blood ammonia. In mouse intervention experiments, rifaximin reduced plasma and hepatic ammonia and alleviated hepatic lipid accumulation by targeting urease-mediated ammonia generation associated with Actinobacteria.^98^ In hepatocyte-based experimental systems, ammonia promoted de novo lipogenesis through the endoplasmic reticulum stress-GCN2-ATF4 axis.^98^ In in vitro tumor-cell systems, glutamine-derived ammonia promoted SCAP dissociation from Insig, activated SREBP-1, enhanced lipid synthesis, and promoted tumor growth.^131^

Microbial hyperammonemia can also arise outside the gut–liver axis in specific clinical settings. Clinical case reports and case series show that *Proteus mirabilis* can cause urinary tract-derived hyperammonemia, particularly in dilated or obstructed urinary tracts, where locally generated ammonia can be reabsorbed into systemic circulation and cause coma.^132^ ^,^ ^133^ In lung transplant cohorts and immunosuppressed patients, *Ureaplasma* spp. and *Mycoplasma hominis* have been identified as microbial triggers of hyperammonemia syndrome.^134^ ^-^ ^136^ These clinical examples indicate that microbial ammonia generation can become systemic when local production is excessive, anatomical barriers are abnormal, or host clearance capacity is insufficient.

### 4.2 Model-specific evidence for local ammonia in intestinal dysmotility

Evidence that increased microbiota-derived ammonia acts as a local compensatory signal remains limited and is currently concentrated mainly in intestinal dysmotility models. In mouse models of intestinal dysmotility, Chen et al. showed that reduced colonic acetylcholine was followed by increased microbial urease activity and local ammonia generation.^75^ In related human constipation samples, similar associations among dysmotility, urease activity, and local ammonia were observed.^75^ In mouse intervention experiments, patient-derived urease-positive strains and engineered urease-expressing bacteria restored colonic acetylcholine and improved intestinal motility.^75^ These findings suggest that, in this specific intestinal context, increased local ammonia may contribute to motility recovery after cholinergic impairment rather than simply represent toxic accumulation.

Experimental physiology studies provide additional support that local ammonia can affect intestinal neuromuscular function. In early ex vivo and in vivo intestinal physiology studies, local ammonia exposure enhanced intestinal movement within a defined experimental range.^76^ This evidence should be interpreted cautiously, because the available data are model-specific and do not establish that increased ammonia is generally beneficial in intestinal disease. Rather, they indicate that the effect of local ammonia depends on exposure range, anatomical site, and the pre-existing functional deficit.

Related host buffering pathways may also shape whether ammonia accumulation becomes damaging or is incorporated into compensatory nitrogen handling. In experimental animal models, hepatic glutamine synthetase acts as an extra-urea-cycle ammonia-clearance route; loss of hepatic GS weakens ammonia clearance, whereas enhancement of hepatic GS increases nitrogen-buffering capacity.^137^ In mouse urea-transporter deficiency models and renal physiology studies, impaired urea transport reduces maximal urinary concentrating ability, supporting a role for urea handling in fluid homeostasis.^138^ ^-^ ^140^ In human nitrogen-balance studies, salvage of exogenous urea nitrogen improved nitrogen balance under marginal low-protein intake. In animal nutritional models, a reshaped gut microbiota enhanced urea nitrogen salvage and helped maintain protein homeostasis under low-nitrogen conditions.^141^ ^-^ ^143^

Together, current evidence supports a restricted interpretation: local ammonia production may participate in motility recovery in selected intestinal dysmotility models. The strongest causal evidence comes from mouse intervention experiments, whereas human constipation data remain mainly associative. These findings should not be generalized to other ammonia-elevated states without evidence for anatomical site, exposure range, cholinergic status, and motility outcome.

### 4.3 Physiological nitrogen recycling and metabolic adaptation

Under specific life stages and nutritional conditions, host-derived urea can be redirected by gut microbes into local nitrogen recycling. Representative physiological examples come from early-life nutrition, low-protein dietary adaptation, and hibernation models. These settings should be interpreted as physiological or adaptive contexts rather than as evidence that ammonia is generally beneficial.

Early life provides a clear example of host-microbe nitrogen recycling. Human milk composition studies showed that urea accounts for a substantial fraction of non-protein nitrogen in breast milk.^18^ ^,^ ^144^ In vitro growth and proteomic studies showed that *Bifidobacterium longum* subsp. infantis can use human milk urea as a nitrogen source and induce urease-related protein expression.^17^In breastfed-infant microbiome samples, urease-related genes are enriched, supporting the relevance of this pathway in early-life gut colonization.^17^ ^,^ ^18^ These findings indicate that, in the infant gut, milk-derived urea can provide nitrogen for urease-positive commensals and may contribute to microbial nitrogen incorporation during early colonization.

Dietary protein restriction provides another model in which microbial nitrogen metabolism participates in host metabolic adaptation. In mouse low-protein diet models, hepatic fibroblast growth factor 21 (FGF21) induction, energy metabolic reprogramming, and white-adipose browning are weakened by antibiotic depletion or germ-free status, indicating microbiota dependence.^111^ ^,^ ^112^ ^,^ ^145^ In a defined-community mouse model, Tanoue et al. further showed that *Bilophila sp. 4_1_30* and *Adlercreutzia equolifaciens* upregulated *nrfA*-related nitrogen metabolism under low-protein conditions, while *Romboutsia timonensis* supported colonization and metabolic output. In that model, co-colonization with wild-type *Bilophila* and *R. timonensis* increased portal ammonia, induced hepatic FGF21, and promoted white-adipose browning through cooperation with the bile acid-FXR axis.^114^ These findings support a low-protein diet-specific microbial ammonia-FGF21 pathway, but its relevance to other dietary or disease contexts remains to be established.

Comparative physiology studies in hibernating animals provide additional evidence for host-microbe urea nitrogen recycling during prolonged nutrient limitation. In hibernating thirteen-lined ground squirrels, gut symbiont-mediated urea nitrogen recycling occurs during prolonged fasting, with increased intestinal urea transporter expression and microbiome urease gene abundance late in hibernation.^146^ Studies in Arctic ground squirrels similarly showed increased incorporation of microbially released nitrogen into host tissues during hibernation, including entry into anabolic amino acid pools.^147^ Subsequent work in thirteen-lined ground squirrels further showed that urea carbon released during microbial ureolysis can enter acetate production, and that acetate can be absorbed and oxidized by the host.^148^ These animal studies indicate that, under prolonged fasting, microbial ureolysis can contribute to both nitrogen and carbon salvage.

At the microbial community level, urea utilization can also intersect with broader carbon–nitrogen metabolism. In genome-resolved and experimental studies of health-associated *Lachnospiraceae*, urease genes and acetate-production-related genes can be co-expressed, and urease-carrying *Blautia* can alter short-chain fatty acid production in the presence of urea.^149^ These findings suggest that microbial urea use can connect ammonium release, ammonia assimilation, short-chain fatty acid production, and cross-feeding within defined community contexts.

Together, evidence from human milk composition studies, infant microbiome samples, in vitro *B. infantis* experiments, mouse low-protein diet models, defined-community mouse models, and hibernating ground squirrel studies supports the role of microbial urea utilization in physiological nitrogen recycling. These findings should be framed as evidence for urea-derived nitrogen reuse under defined physiological or dietary conditions, not as evidence that reduced ammonia generally causes disease or that increased ammonia production is broadly beneficial across host states.

### 4.4 Possible effects of reduced microbial ammonia-derived nitrogen input

Evidence for insufficient microbiota-derived ammonia as a disease contributor is currently more limited than the clinical evidence for hyperammonemia. The strongest support comes from selected preclinical models rather than from broad human disease cohorts. In a chronic stress mouse model, stress altered the abundance distribution of urease-positive gut bacteria, reduced blood ammonia, decreased brain glutamine availability, impaired GABAergic signaling, and increased stress susceptibility. Pharmacological inhibition of intestinal urease further reduced fecal and blood ammonia and aggravated depression-like behavior, whereas supplementation with the representative urease-positive bacterium *Streptococcus thermophilus* partly reversed the behavioral abnormality.^9^ These findings are consistent with a model-specific link between microbial urease activity, basal ammonia-derived nitrogen input, brain glutamine metabolism, and stress-related behavior.

This interpretation is biologically plausible because the brain glutamine–glutamate–GABA cycle requires continuous nitrogen input. Astrocytic glutamine synthetase converts ammonia and glutamate into glutamine, which then supports neuronal synthesis of glutamate and GABA.^4^ ^,^ ^150^ Experimental and clinical studies of glutamine synthetase deficiency further show that impaired glutamine synthesis can cause severe neurological dysfunction, including neonatal epileptic encephalopathy and abnormal brain development.^151^ ^-^ ^155^ In stress-related models, local glutamine deficiency in the prefrontal cortex can induce depression-like behavior, whereas glutamine supplementation or activation of glutamine synthetase can improve stress-related behavioral and neurochemical abnormalities.^156^ ^-^ ^160^ These studies do not prove that low microbial ammonia is a general cause of neurological disease, but they support the idea that reduced nitrogen input from microbial urease activity may affect brain function in specific experimental contexts.

Other downstream nitrogen carriers, including arginine and citrulline, may also help interpret ammonia-related metabolic states, but the evidence linking them directly to insufficient microbiota-derived ammonia remains indirect. Arginine deficiency and impaired nitric oxide production contribute to vascular and neurological complications in urea cycle disorders such as argininosuccinate lyase deficiency.^161^ ^-^ ^164^ Low plasma citrulline is widely used as a marker of reduced small-intestinal epithelial mass and impaired absorptive function in intestinal failure, short bowel syndrome, villous atrophy, and radiation-induced intestinal injury.^165^ ^-^ ^169^ These examples indicate that downstream nitrogen carriers can reflect organ-specific nitrogen metabolic impairment, but they should not be interpreted as direct evidence that reduced microbial ammonia flux is broadly pathogenic.

Together, these findings support an emerging, model-specific hypothesis that reduced microbial nitrogen input can affect downstream nitrogen carriers such as glutamine, arginine, and citrulline. At present, this hypothesis is best supported for the link between urease-positive bacteria, ammonia-derived nitrogen, and brain glutamine in chronic stress models, whereas its relevance to other diseases and human populations requires further validation.

**Table 1.** Context-specific host responses to gut microbial ammonia.

Physiological or disease context	Ammonia-related readout - site and comparator	Microbial feature or pathway implicated	Host interface or functional interpretation	Evidence source and interpretive strength	References
Cirrhosis/hepatic encephalopathy	Peripheral or plasma ammonia and fecal urease activity are elevated relative to healthy or non-HE controls; brain glutamine is increased in HE-associated settings.	Enrichment of urease-positive taxa, proteolytic fermentation, amino acid deamination, and selected oral-to-gut strain overlap.	Gut-derived ammonia contributes to systemic hyperammonemia when hepatic first-pass clearance is impaired and portosystemic shunting is present; systemic ammonia then affects astrocyte osmotic balance, glutamine-glutamate cycling, and neurological function.	Strong clinical and mechanistic support for elevated systemic ammonia; microbial contribution is supported but acts together with hepatic clearance failure and shunting.	[1,3,4,123–126,151,170]
Intestinal dysmotility/constipation	Colonic or fecal ammonia and urease activity are locally increased relative to dysmotility or control comparators; systemic hyperammonemia is not the main readout.	Urease-positive bacterial activity; patient-associated or engineered urease-expressing bacteria in mouse intervention models.	Local ammonia was associated with acetylcholine-linked ENS signaling and improved intestinal transit in dysmotility models.	Human association plus mouse intervention evidence; model-specific and not generalizable to all ammonia-elevated intestinal states.	[75–77]
Low-protein dietary adaptation	Portal venous ammonia is increased in defined-community low-protein mouse models relative to germ-free, antibiotic-depleted, or comparator colonization states.	*nrfA*-associated reductive nitrogen metabolism, microbial ammonia generation, and urea-nitrogen salvage in defined microbial communities.	Under low-protein conditions, microbiota-derived portal ammonia can contribute to hepatic FGF21 induction and adipose-tissue metabolic adaptation.	Germ-free and defined-community mouse evidence; diet-specific adaptive pathway.	[111,114]
Systemic hyperammonemia-associated skeletal muscle dysfunction	Peripheral or systemic ammonia is elevated in cirrhosis or experimental hyperammonemia models relative to controls.	Not a specific microbial pathway; gut-derived ammonia may contribute to the systemic ammonia pool in liver disease.	Elevated systemic ammonia increases skeletal-muscle ammonia-buffering demand and promotes muscle atrophy through mitochondrial stress, oxidative injury, autophagy, and myostatin-related pathways.	Human disease association and animal hyperammonemia models; muscle phenotype is not microbial-source specific.	[107,109,110]
Alcohol-associated fatty liver disease	Plasma and hepatic ammonia are increased after alcohol exposure relative to control conditions.	Gut-derived ammonia generation and urease-associated microbial activity; rifaximin-sensitive microbial contribution in experimental models.	Gut-derived ammonia can facilitate ethanol metabolism and activate ATF4-dependent de novo lipogenesis, thereby promoting alcohol-associated hepatic steatosis.	Human samples plus mouse intervention evidence; disease-specific microbial ammonia pathway.	[98]
Multiple myeloma-related bone disease	Circulating ammonium is increased in disease or *E. cloacae*-associated models relative to controls.	*Enterobacter cloacae*-associated *dcd*/*nagB*-related ammonium production and non-urease nitrogen transformation.	Microbial ammonium production promoted osteoclastogenesis and bone disease phenotypes in experimental models; human evidence remains associative.	Human cohort association plus animal and cell-based disease models; context-specific pathological pathway.	[52]
Chronic stress susceptibility	Peripheral blood ammonia and brain glutamine are reduced relative to non-stressed mice in a chronic stress model.	Reduced abundance or activity of urease-positive bacteria; altered basal gut-derived ammonia availability.	Reduced microbial urease-derived nitrogen input was linked to reduced brain glutamine and altered GABA-related stress responses.	Mouse model evidence only; supports a model-specific low-ammonia hypothesis and should not be generalized to human disease.	[9,171]
Early-life nitrogen salvage	Milk urea-derived nitrogen is incorporated into microbial nitrogen pools; pathological ammonia accumulation is not the primary readout.	Infant-associated urease-positive or urea-utilizing microbes, including urea use by *Bifidobacterium*-associated pathways, and microbial ammonia assimilation.	Host-derived or milk-derived urea can be hydrolyzed and reincorporated into microbial amino acids and biomass during early colonization.	Human milk and infant microbiome data plus in vitro microbial physiology; physiological nitrogen-recycling context.	[6,17,18]

## 5 Clinical translation and therapeutic strategies targeting microbial ammonia metabolism

Clinical translation should focus first on settings in which excessive ammonia production, impaired hepatic clearance, portosystemic shunting, or reduced extrahepatic buffering leads to systemic hyperammonemia. In liver disease and overt hyperammonemia, reducing intestinal ammonia generation remains a clinically established goal. Lactulose and rifaximin remain standard therapies for hepatic encephalopathy and reduce systemic ammonia burden through effects on colonic pH, transit time, microbial activity, and ammonia absorption.^151^ ^,^ ^170^ More targeted approaches should be interpreted according to the dominant source of ammonia and the strength of supporting evidence. Direct inhibition of microbial urease is mechanistically supported by liver injury models and by clinical associations between fecal urease activity, plasma ammonia, and overt hepatic encephalopathy risk in cirrhosis.^22^ ^,^ ^123^ However, urease inhibition should not be assumed to cover all ammonia-producing states, because non-urease nitrogen-transforming reactions may also contribute to ammonia accumulation in specific disease contexts.^52^

Enhancing microbial ammonia assimilation is a second potential strategy, but it remains investigational. Engineered bacterial systems, including *E. coli Nissle* 1917 designs that channel ammonia into arginine biosynthesis, can reduce systemic ammonia burden in hyperammonemic animal models.^172^ ^,^ ^173^ However, SYNB1020 showed safety and detectable in vivo metabolic activity in healthy volunteers but did not establish stable blood-ammonia lowering in patients with cirrhosis.^173^ These findings support engineered ammonia assimilation as a preclinical or early clinical direction rather than an established ammonia-targeted therapy.

Dietary intervention should be framed as substrate management rather than as direct ammonia therapy. Microbial ammonia production is influenced by protein load, microbially accessible carbohydrate availability, luminal urea input, transit time, pH, and the overall fermentation background.^23^ ^,^ ^174^ In proteolysis-dominant settings, reducing excessive protein substrate and increasing microbially accessible carbohydrates may reduce colonic ammonia generation and related protein fermentation products.^23^ ^,^ ^175^ However, dietary studies often change multiple components simultaneously, including protein amount, carbohydrate availability, fat content, micronutrients, and total substrate structure. Therefore, observed effects should be interpreted as consequences of the overall dietary substrate pattern, not attributed narrowly to protein quality alone. In cirrhosis and hepatic encephalopathy, unnecessary protein restriction should be avoided because it can worsen malnutrition, promote skeletal muscle breakdown, and weaken peripheral ammonia buffering.^176^ ^-^ ^178^

Overall, ammonia-targeted translation should prioritize established hyperammonemic settings and should avoid extrapolating model-specific findings into general clinical strategies. Approaches aimed at preserving local ammonia-derived nitrogen input, reshaping complex microbial communities, or improving cognitive outcomes through broad microbiota remodeling should currently be treated as research hypotheses rather than mature therapeutic concepts.

Measurement should be kept as a practical checklist rather than a separate methodological framework. A minimum reporting set should include sampling compartment, sample handling, total ammonia concentration, blood urea or blood urea nitrogen, luminal or fecal urea when available, pH, and NH_3_/NH_4_^+^ speciation when possible (Figure 3).^1^ ^,^ ^60^ ^,^ ^90^ ^,^ ^120^ ^,^ ^179-181^ Sampling compartments should be specified more precisely than fecal or intestinal and, where feasible, should distinguish ileal luminal or ileostomy/ileal effluent, ascending or proximal colonic, distal colonic, fecal, mucus-associated, portal, and peripheral blood compartments. Functional interpretation should combine ammonia measurements with fecal or luminal urease activity, urease genes, candidate deamination-related genes, ammonia-assimilation genes, liver and kidney function, portosystemic shunting, barrier status, inflammation, and muscle mass. Blood urea or blood urea nitrogen should be included because it reflects the circulating host-derived substrate pool available for delivery to ileal and colonic compartments for microbial ureolysis, although it should not be interpreted as a direct measure of microbial ammonia production.^12^ ^,^ ^182^ ^,^ ^183^ This restricted measurement panel is intended to reduce overinterpretation and improve comparability across microbiome, animal, and clinical studies.

## 6 Knowledge gaps and future directions

Several gaps limit mechanistic interpretation of gut microbiota-derived ammonia metabolism.

First, physiological ranges and pathological thresholds of ammonia across spatial layers remain poorly defined. Peripheral blood ammonia has clear clinical value in cirrhosis and hepatic encephalopathy and can support risk assessment and prognostic evaluation. However, it primarily reflects an integrated systemic readout after ammonia has entered the circulation and does not directly represent exposure in the intestinal lumen, mucus layer, portal blood, or neural tissue.^1^ ^,^ ^120^ ^,^ ^184^ Future studies should therefore establish spatially resolved measurement systems that distinguish local ammonia-derived nitrogen use in defined physiological or preclinical models, portal-level ammonia exposure, and systemic ammonia burden that exceeds host buffering capacity.

Second, strain- and pathway-level maps of microbial ammonia metabolism remain incomplete. The field has begun to move from asking which bacteria change to asking which functional programs are activated, but it remains unclear which urease operons, deamination pathways, *nrfA*-dependent nitrate/nitrite reduction to ammonium, and ammonia-assimilation genes dominate in vivo nitrogen flow in specific disease states.^7^ ^,^ ^52^ ^,^ ^114^ Future work should distinguish the chemical origins of ammonia, including urea hydrolysis, protein and amino acid deamination, nucleotide or amino sugar metabolism, oxidized-nitrogen reduction, and host-microbe urea-nitrogen recycling. Integrating ammonia-generating programs, assimilation capacity, substrate supply, and community cross-feeding within a single analytical framework will be necessary to determine whether ammonia is locally fixed or converted into epithelial, portal, or systemic exposure.

Third, causal evidence requires further strengthening. Many studies still rely on correlations among microbiota composition, functional genes, and ammonia concentration. The next phase of the field will require stable isotope tracing, germ-free or defined-community models, strain-level functional validation, microbial engineering, and longitudinal intervention studies.^6^ ^,^ ^8^ In particular, three evidence layers should be separated: microbiota or metabolic features associated with ammonia, microbial pathways that regulate ammonia flux, and host pathological or adaptive phenotypes driven by ammonia itself. Establishing such an evidence hierarchy will help prevent ammonia from being treated as a passive accompanying marker or overinterpreted as pathogenic without mechanistic validation.

Fourth, standardization of ammonia measurement should become a methodological priority. Future studies should use consistent protocols for spatial sampling, synchronized pH recording, sample processing, NH_3_/NH_4_^+^ speciation, functional gene detection, enzyme-activity assays, and isotope flux analysis.^1^ ^,^ ^181^ ^,^ ^184^ In microbiome studies, a single fecal ammonia or peripheral-blood ammonia measurement is insufficient to define the ammonia exposure. More informative designs should simultaneously trace ammonia source, local chemical environment, microbial functional state, and host clearance capacity. Only when ammonia measurement advances from crude concentration comparison to spatially resolved, functionally annotated, and flux-oriented analysis will multicenter comparison and clinical stratification become reliable.

From a study-design perspective, the next generation of high-quality work should meet several minimum criteria. First, studies should use spatially stratified sampling whenever feasible, rather than relying solely on a single fecal or peripheral-blood readout. Second, ammonia measurement should be paired with pH, substrate supply, functional genes, enzyme activity, and metabolic flux, rather than treating microbial abundance or functional gene abundance as direct proxies for flux. Third, animal and human intervention studies should clearly distinguish intervention goals, including reducing ammonia generation, enhancing assimilation, promoting excretion, and improving host buffering reserves. Fourth, longitudinal designs should be used to track dynamic relationships among microbial nitrogen metabolism, ammonia exposure, and clinical outcomes, rather than relying on cross-sectional association alone. Such designs would make the concept of ammonia as a context-dependent mediator testable, rather than merely descriptive.

## 7 Conclusions

Gut microbiota-derived ammonia is best interpreted through microbial production, microbial assimilation, anatomical exposure, and host clearance capacity. Elevated systemic ammonia remains a well-established pathological driver in liver disease and hyperammonemia, whereas adaptive ammonia use or reduced ammonia-derived nitrogen input is supported mainly by context-specific evidence from selected physiological and preclinical models. The same microbial ammonia-producing pathway may therefore have different consequences depending on whether ammonia is incorporated into microbial biomass, retained in the intestinal lumen, delivered to the portal vein, or released into systemic circulation.

Future studies should combine spatial sampling, microbial pathway analysis, pH and urea measurements, isotope tracing, and host clearance assessment to define ammonia flux in specific disease settings. This approach will help distinguish excessive ammonia production, insufficient assimilation, increased epithelial or portal exposure, impaired hepatic clearance, and reduced host buffering capacity, thereby providing a more precise basis for microbiome-based intervention.

## Data Availability

No primary datasets were generated or analyzed in this manuscript.
